# Mapping the feel of the arm with the sight of the object: on the embodied origins of infant reaching

**DOI:** 10.3389/fpsyg.2014.00576

**Published:** 2014-06-11

**Authors:** Daniela Corbetta, Sabrina L. Thurman, Rebecca F. Wiener, Yu Guan, Joshua L. Williams

**Affiliations:** ^1^Director Infant Perception-Action Laboratory, Department of Psychology, The University of TennesseeKnoxville, TN, USA; ^2^Department of Psychology, The University of TennesseeKnoxville, TN, USA; ^3^Department of Psychology, Armstrong State UniversitySavannah, GA, USA

**Keywords:** reaching, eye-tracking, human infants, visuo-motor mapping, embodiment, longitudinal study, skill emergence, object-directed visual attention

## Abstract

For decades, the emergence and progression of infant reaching was assumed to be largely under the control of vision. More recently, however, the guiding role of vision in the emergence of reaching has been downplayed. Studies found that young infants can reach in the dark without seeing their hand and that corrections in infants' initial hand trajectories are not the result of visual guidance of the hand, but rather the product of poor movement speed calibration to the goal. As a result, it has been proposed that learning to reach is an embodied process requiring infants to explore proprioceptively different movement solutions, before they can accurately map their actions onto the intended goal. Such an account, however, could still assume a preponderant (or prospective) role of vision, where the movement is being monitored with the scope of approximating a future goal-location defined visually. At reach onset, it is unknown if infants map their action onto their vision, vision onto their action, or both. To examine how infants learn to map the feel of their hand with the sight of the object, we tracked the object-directed looking behavior (via eye-tracking) of three infants followed weekly over an 11-week period throughout the transition to reaching. We also examined where they contacted the object. We find that with some objects, infants do not learn to align their reach to where they look, but rather learn to align their look to where they reach. We propose that the emergence of reaching is the product of a deeply embodied process, in which infants first learn how to direct their movement in space using proprioceptive and haptic feedback from self-produced movement contingencies with the environment. As they do so, they learn to map visual attention onto these bodily centered experiences, not the reverse. We suggest that this early visuo-motor mapping is critical for the formation of visually-elicited, prospective movement control.

## Introduction

Reaching for objects is a fundamental skill that emerges in infancy around 3–5 months of age. Understanding how this skill forms and develops has been a core area of study in developmental psychology since the 1930s (Halverson, 1931; Piaget, 1936/1952; Gesell and Amatruda, 1946). Indeed, the onset of object-directed reaching marks an important transition in the development of infants' voluntary activity and provides essential foundations for the development and refinement of future motor, perceptual, and cognitive behaviors (Bushnell and Boudreau, 1993; von Hofsten, 1993, 2009). Despite extensive research in this area, the process by which infants learn to bring their arm in contact with a wanted object is still open to much investigation. For the longest time, the emergence of reaching was thought to be under the control of visual guidance. It was assumed that infants needed to see their hand in order to steer it toward the target (e.g., White et al., 1964; von Hofsten, 1979; Bushnell, 1985). But in recent decades, researchers have begun to question the guiding role of vision for the emergence of infant reaching. Some demonstrated that from their earliest attempts, infants can reach in the dark toward a glowing target without seeing their hand (Clifton et al., 1993). This suggested that infants rely primarily on proprioceptive information, not vision, to begin controlling and directing their arm toward a specific location in space (Thelen et al., 1993; Robin et al., 1996). As a result, recent accounts have begun to emphasize a more embodied process of learning to reach in contrast to the visually-guided approach that has dominated the field for several decades.

This shift toward an embodied account of learning to reach, however, leaves some questions unanswered regarding the actual role of vision in the emergence of infant reaching and particularly how vision and action map onto each other. We know that in daily, lighted surroundings, when vision is available for reaching, infants do fixate the objects (McCarty and Ashmead, 1999). They do so even weeks before reaching onset (von Hofsten, 1984). Specifically, when an object is within arm's reach and being fixated, pre-reaching infants already begin to display object-oriented changes in their arm and hand movements compared to when they are not fixating the object (von Hofsten, 1984) or to when objects are not present (Bhat et al., 2005). Finally, despite clear evidence that infants can purposefully reach for a glowing object in the dark without seeing their hand (Clifton et al., 1991, 1993), studies have revealed an effect of vision on the formation of goal-directed movements when target or arm are occluded (Clifton et al., 1994; McCarty and Ashmead, 1999; Pogetti et al., 2013). Thus, this body of work suggests that eye and hand interact with one another well before the emergence of purposeful reaching, and continue to do so afterwards. What remains unclear is how looking at the object and bringing the hand to that location occurs at first when infants perform their initial intentional attempts to hit the target. What visuo-motor mapping process allows this to happen?

The goal of this paper is to examine anew the role of vision in relation to the emergence of goal-directed reaching in infancy, particularly in light of the more recent embodied accounts on learning to reach. We ask how do infants figure out how to map the feel of their arm to a specific location identified visually if infants' first reaching attempts are mainly controlled proprioceptively? Does vision provide any specific information in this process *prior* to reaching onset that could help tune infants' arm movements to the target location? Does proprioceptive control of the arm, from reach onset, improve such that infants become increasingly more successful and more accurate at bringing their hand toward the object area attended visually? Such scenario would be in line with our current understanding of the early process of learning to reach. It presupposes that vision is prospective and that progression in the development of reaching is a matter of learning how to improve movement control to align the movement endpoint to the visually attended target area. But could it be the other way around, that vision is mapping onto the proprioceptive movement experience of the infant? This other scenario would offer a more consistent embodied account of learning to reach by assuming that the use of vision for the control of future-oriented actions could possibly originate from infants' initial and self-produced proprioceptive movement experiences. A third scenario could also be that vision and action map onto one another in a more reciprocal fashion. This paper aims to examine these hypotheses on the developmental origins of object-directed visuo-motor mapping in infancy. We first review how previous research on infants learning to reach has addressed the question of perceptual-motor mapping. Then, we present preliminary, first-time longitudinal data on object-directed looking (captured via eye-tracking) and reaching in three infants that we followed weekly throughout the transition to reaching, to examine how the above scenarios play out. We attempt to gain insights on the process underlying the formation of visuo-motor mapping at reach onset (1) by identifying whether looking patterns at the target objects prior to the onset of reaching can help predict the formation of early goal-directed movement, (2) by tracking whether these looking patterns at the object change in the weeks following reach onset, and (3) by examining if there is some spatial correspondence between the history of looking patterns at the object and the history of point of hand-object contact after reach onset that could support one of the suggested scenarios. As we will show, these preliminary data further extend previous embodied accounts of infants learning to reach. They suggest the possibility that mapping the feel of the hand with the sight of the object occurs by learning to align visual attention to the point of first hand-object contact, and not the reverse, as previously thought. We discuss the implication of these findings for the development of prospective control from an embodied perspective.

### Learning to reach from a visually-guided account

Traditional accounts on the development of infant reaching greatly emphasized the role of vision in the process of guiding the hand toward the target. Piaget (1936/1952) was the first to describe this visually-guided process from observing his children. He reported that the emergence of reaching was elicited by the simultaneous perception of the hand and object in the same visual field. From that point, infants actively learned to match the sight of their hand to the sight of the object by coordinating two initially isolated schemes—the one for looking and the one for grasping. This combined scheme reflected a new level of functioning between vision and action, and marked the naissance of goal-directed actions. This view, that vision of the hand and target were critical for the emergence of infant reaching, was later heralded by a number of studies.

White et al. (1964) described the developmental steps leading to the emergence of visually-guided reaching by following infants longitudinally in a state hospital over their first 6 months of life. They reported several occurrences of infants alternating glances between hand and object in the months preceding reach onset. At reach onset, they noticed that these glances were used to guide the hand to the object. However, in the following weeks, they indicated that these glancing patterns dropped fairly rapidly and infants were able to lift their arm quickly from out of view to reach for the target. Assumingly, a more direct visuo-motor match had formed after a few months of visually-guided practice.

Subsequent studies recorded the kinematics of infants' reaching trajectories. They found that infants' early reaching trajectories were poorly controlled and contained many corrections and changes in direction before the hand attained the target (von Hofsten, 1979, 1991). Such indirect trajectories were interpreted as in line with the visually-guided reaching hypothesis, that vision was needed initially to actively steer the hand step-by-step closer to the target. Some studies even manipulated vision by using mirrors and displacement prisms to perturb infants' eye-hand coordination during reaching (McDonnell, 1975, 1979; Lasky, 1977). Results indicated that only older infants were affected by the mirrors/prisms. Researchers concluded that young infants did not experience a disruption in perceptual-motor coordination because they were visually monitoring their displaced hand in relation to the displaced target through the prisms, which was considered in support of the visually-guided hypothesis.

In sum, these earlier studies agreed that infants learned to reach via a top-down, visually-guided process, as if the mind was “teaching” the hand where to move in space to contact the target. Visually-guided reaching declined after months of intensive practice and gave way to visually-elicited reaching assuming a more direct spatial match between felt arm and seen object (Bushnell, 1985).

### Learning to reach from an embodied account

Today, researchers agree that learning to reach toward a wanted target is a protracted process that involves much practice over many months before infants can perform smooth and fully adapted movement patterns (Thelen et al., 1996; Konczak and Dichgans, 1997; Corbetta and Snapp-Childs, 2009). However, findings from these recent decades disagree with the premises that vision and action are separated and need to be coordinated through visual guidance in order to develop goal-directed reaching. Two lines of work contributed to this change in view.

Clifton and colleagues (Perris and Clifton, 1988; Clifton et al., 1991, 1994) found that infants can reach in the dark toward glowing or sounding objects without seeing their hand. Further, they investigated whether not seeing the hand would delay the emergence of reaching (Clifton et al., 1993). They followed infants weekly for a month prior to the onset of reaching. They found that infants who were presented with glowing objects in the dark over the weeks began to reach at approximately the same time as infants who were presented with objects in the light. This confirmed that vision of the hand was not needed to direct it to a specific spatial location even at reach onset.

The second line of studies related to trajectory formation and the circuitous hand paths typical of infants' early reaches. Thelen et al. (1993, 1996) tested four infants weekly in standard lighted conditions through the transition to reaching and subsequently throughout the end of their first year of life. They found that the initial distortions in hand trajectory were not the result of visual guidance of the hand, but rather the product of infants' inability to adequately calibrate the speed of their arm movements to the desired goal (see also Konczak et al., 1995). For example, when infants produced reaching movements with excessive speed, important motion dependent forces were generated throughout the joints and segments of the arm, which in turn acted as internal perturbations to movement coordination and contributed to drag the hand away from its intended goal. In order to counteract these disruptive forces and attain the object, infants needed to break these forces in movement and steer their hand toward the target, thus causing the observed changes in trajectory. Breaking of the movement speed and steering of the hand was not done by visual control, because infants continued to fixate on the target during this process. It was accomplished by modulating muscle forces. In subsequent weeks, as infants continued to practice reaching, they began to alter the speed of their reaching movements, suggesting that they were attempting to figure out how to calibrate their movement speed to the intended goal. This revealed an embodied learning process that involved many trials and errors, through which infants proprioceptively experienced a wide range of movements, some fast, some slow, thereby testing the dynamic boundaries of their movement in relation to the goal. Infants learned to map their intrinsic movement dynamics to the intended target goal by remembering the ones that led to good outcomes, and increasingly selecting these good solutions in the production of future attempts (Sporns and Edelman, 1993; Thelen, 1995).

These newer lines of work indicated that infants do not learn to reach via a top-down process where the mind commands the body, but rather do so by controlling the proprioceptive feel and intrinsic dynamics of their arm movement in relation to a goal located in space. This is a deeply embodied dynamic process in which mind and body work in concert, and in which a more exact mapping between intentions and arm movement forms through repeated sensory-motor experience, producing a behavior that becomes increasingly direct and tuned to its intended goal (Chiel and Beer, 1997; Corbetta, 2009).

### The missing link: mapping the feel of the hand with the sight of the object

What remains unclear from this prior body of work is how infants discover how to meet their intentions by mapping the proprioceptive sensations of their moving arm to a visually detected location in space. When beginning to reach, and reproducing this behavior, infants display a new intentional skill never performed before. How does looking at the object (even if performed in the dark toward a glowing object) and bringing the hand in that specific location come together in the first place?

The embodied accounts reviewed above have somewhat downplayed the critical role of vision for learning to reach despite abundant evidence indicating that visual input matters for reaching. As mentioned earlier, when infants are approaching reach onset, they fixate the target object intensely (von Hofsten, 1984, 1986). They continue to do so at reach onset and thereafter while improving arm control (Williams, 2009, 2011). Blind infants, who cannot build visual experience from birth, develop reaching at a later age (Bigelow, 1986; Troester and Brambring, 1993). Additionally, a large literature supports the prospective role of vision in the planning and execution of future-oriented actions (Jeannerod, 1988). Adult studies that used eye-tracking in the context of goal-directed movement activities have shown that the eyes usually precede the action; they aid selecting ahead of time the location of the action, but also (among other things) where and how the action should occur (Land et al., 1999; Johansson et al., 2001; Horstmann and Hoffmann, 2005; Rosander and Von Hofsten, 2011). Such prospective control of vision has been documented in infants reaching as well, for example, for identifying objects' spatial locations, (Morrongiello and Rocca, 1989), for picking up object-related information (Lockman et al., 1984; von Hofsten and Fazel-Zandy, 1984; Witherington, 2005; Berthier and Carrico, 2010), intercepting moving objects (von Hofsten, 1983; Rosengren et al., 1988), and adjusting movement in precision tasks (Carrico and Berthier, 2008; Berthier and Carrico, 2010). Vision was even found important to stimulate infants' motivation to develop active search strategies (Bojczyk and Corbetta, 2004). Such work, however, contrasts with other findings suggesting that the use of vision for movement planning and execution in infancy does not occur before 6 months of age (Berthier and Carrico, 2010) and may even continue to develop until the second year of life, especially in precision tasks (Carrico and Berthier, 2008). This raises the question of how infants learn to map the feel of their arm with the sight of the target. Indeed, it is not known if at reach onset infants control the proprioceptive feel of their arm to approximate a spatial location that is visually defined, or, if it could be the other way around, that infants map their visual attention to their proprioceptive movement experience; or maybe even a combination of both, that is, vision and proprioception are mapping onto each other. Given the reviewed evidence, it seems critical to reevaluate the role of vision in the formation of early goal-directed movements, particularly around the emergence of reaching.

In this paper, we focus on the period around reaching onset to address three goals. Prior to reach onset, we investigate whether infants simply visually attend the location of the object without specific pattern of visual exploration of the object *per se*, or whether they already examine the shape or physical properties of object in certain ways, casting the possibility of a pre-nascent visual selective process in preparation for learning to reach. We examine how infants' object-directed visual behaviors develop following reach onset, when rapid changes in arm control are taking place. Additionally, by analyzing object-directed visual attention throughout the transition to reaching, we aim to gain new insights into the visuo-motor mapping process that underlies the emergence of infant reaching. Based on the existing literature, we see three possible scenarios that could account for how vision and action may come together when infants begin to reach for an object purposefully, for the first time. We present these scenarios first and then evaluate them against preliminary longitudinal data on the looking and reaching behaviors of three infants over an 11-week period.

### Possible scenarios and predictions

- *Scenario 1 (or the prospective control hypothesis)*. This scenario assumes that vision dictates where the hand needs to go (a sort of visually-elicited hypothesis). This would imply that infants learn to map the feel of their arm onto the sight of the object by learning to control their arm increasingly accurately to bring it where visual attention on the object is directed. Predictions from this scenario would imply that a visual selection process or particular pattern of fixations at the object could possibly form and become more observable in the weeks preceding reach onset. At reach onset and in subsequent weeks, as infants increase proprioceptive control of their arm, they would gradually figure out how to better align the transport of their hand to the vicinity of the object area visually attended. The developmental origins for such a visuo-motor mapping process can be found in the initial eye-hand coordination and pre-reaching skills of newborns (von Hofsten, 1982; van der Meer et al., 1995b), but also in their developing predictive visual abilities (von Hofsten, 1980, 2005; van der Meer et al., 1994, 1995a). Such early prospective control abilities and initial eye-hand coordination could strengthen in the weeks preceding reach onset and facilitate the transition to reaching. As object-directed vision would become more selective prior to reach onset, infants' intents toward the object could also increasingly materialize. Initially, such intention could be instantiated by the use of poorly controlled arm movements and rapid, inaccurate swipes at the target. But with rapid improvement in proprioceptive arm control infants would become increasingly capable of nearing and even matching with their hand the visually pre-selected spatial location. In this scenario, movement would align to vision.
- *Scenario 2 (or the embodied account hypothesis)*. This scenario would assume that infants can take advantage of their proprioceptive experience prior to reach onset to discover how to direct their arm to a specific location. Vision would become calibrated to the action as the result of accidental events. Early in life, infants move their arms around in seemingly unintentional ways. Doing so, they can hit objects inadvertently, without seeing them, but receive immediate haptic and proprioceptive feedback about the posture and location of their arms and hands in space. Such accidental events could eventually cause the infants to direct their regard toward the location where hand-object contact occurred. Hence, vision could begin to be associated to the arm movement experience of the child as a consequence of the haptic and proprioceptive feedback received. After many repetitions of such events, a basic form of movement intentionality could emerge when the feel of an arm movement in a given direction would happen to match the sight of an object in that same direction, but the experience would still be proprioceptively-guided, not visually-elicited. As vision would continue to map onto these emergent, successful, proprioceptively controlled goal-directed movements, a more accurate selective or prospective role of vision could develop, mainly as a consequence of connecting what is seen to what is felt, not the reverse. Predictions from this scenario would entail that infants can demonstrate a relatively accurate sense of their arm projection in space at reach onset. We would expect little or no specific pre-selective looking patterns at the object in the weeks preceding the emergence of reaching. Over time, we should see increased visual attention directed toward where the hand is reaching as a result of infants discovering how to align vision to their actions and beginning to anticipate where to bring their arm in space. This sensory-motor scenario can be rooted in infants' early sense of their own body and movements (Rochat, 1998; Rochat and Morgan, 1998). Neonates respond to touch and orient themselves in a sophisticated manner from the first days of life (Rochat and Hespos, 1997). In subsequent months, infants continue to discover the action possibilities of their body through movement explorations in time, space, and self-perception that are not necessarily goal-directed. By doing so, they learn to detect contingencies between their felt actions and the interesting visual outcomes they may cause and observe (Angulo-Kinzler, 2001), including relating arm movements to object touch, even if they occurred accidentally at first and without looking directly. These early movement experiences and contingencies could contribute to the formation of new action patterns increasingly associated with a stronger and more accurate sense of limb movements in space without needing explicit visual guidance. This would be consistent with studies on infant reaching in the dark, even for unseen, auditory perceived objects (Perris and Clifton, 1988; Clifton et al., 1991). Furthermore, such scenario, where vision is being mapped onto action, would be compatible with some current embodied mind views. Through early bodily experiences, infants could form extended space representations of their actions (Borghi et al., 2013), “mesh” them with visual experiences and varied contexts, and remember them for future actions (Glenberg, 1997), and thus, develop a cognition for action that would initially be deeply body based (Wilson, 2002).
- *Scenario 3 (co-mapping of sight and feel)*. This third scenario would correspond to a mix of the two described above and would not assume any dominance of vision over proprioception (scenario 1), or proprioception over vision (scenario 2), but would rather cast the emergence of reaching as the product of a continuous process where both prospective vision and proprioceptive feel of the arm experienced in the month before reach onset become progressively integrated. Predictions should show that both sight of the object and feel of the arm are increasingly mapped onto each other, but are not related to particular visual looking trends prior to reaching onset, nor to any movement tendency after reach onset.

To examine the plausibility of these scenarios, we documented the looking (captured via eye-tracking) and reaching patterns of three infants that we began to see from the age of 2–2.5 months old (that is prior to the emergence of reaching). We followed them until they were 12 months of age, but for the purpose of this report we focus only on an 11-week period around the transition to reaching. Each week, infants were presented with 3D objects that they could visually scrutinize for up to 5 s before they would be allowed to reach for them. Infants were presented with five kinds of objects. Here we describe in detail the results related to a drumstick-shaped object (a sphere attached to the end of a rod) and contrast them with those of a plain rod with no distinct features. For each week, we report how looking patterns were distributed on the objects. When infants began to reach, we documented where they brought their hand to make the first contact with the object and related it to the looking patterns. We also compared their performance to a group of 9-month-old infants tested in the same conditions. Because 9-month-olds have more reaching experience and demonstrate decent prospective control in reaching (Lockman et al., 1984; von Hofsten and Fazel-Zandy, 1984; Piéraut-Le Bonniec, 1985; Bloch, 1988; von Hofsten and Rönnqvist, 1988), they constitute a good developmental norm.

## Methods

### Participants

Eighteen infants participated in this study. Fifteen of them (6 females) were 9 months old (±1 week) at the time of testing. They were seen only once and their data were used in this report to provide a developmental reference norm. The other three infants (2 females) were followed longitudinally from about 2 months of age, and up to the end of their first year of life. This report presents the 11-week period around the transition to reaching (that is, 5 weeks prior to reaching onset, the week of reaching onset, and 5 weeks following reaching onset). Table 1 summarizes the ages (in weeks) at which we obtained useable eye-tracking data and when reach onset occurred. For infant MC, week 10 was used in replacement for missing data at week 11. Infant ME only provided useable eye-tracking data prior to reaching at week 20. Infant AC had missing data at weeks 11 and 12 prior to reach onset. All infants were recruited from the Greater Knoxville, Tennessee area (USA), via formal mailings, follow-up phone calls, or various forms of personal contact. Parents voluntarily enrolled their infants in the study and informed consent was collected for all infants. Infants were born full term, and were free of visual or motor impairments. All participating infants were White, except for one longitudinal infant who was African American. Parents were given $10 and a photograph of their child at each visit, and received a certificate of participation.

**Table 1:** **Ages (in weeks) for the three longitudinal infants when tested over the 11-week period**.

**Infant ID**	**Weeks prior reach onset**					**Reach onset**	**Weeks after reach onset**				
MC	10	12	13	14	15	**16**	17	18	19	20	21
ME	-	-	-	-	20	**21**	22	23	24	25	26
AC	10	-	-	13	14	**15**	16	17	18	19	20

*Weeks of reach onset are marked in bold. A hyphen indicates that no useable data were collected on that particular week*.

### Materials

Testing sessions were completed in a well-lit room. A custom-designed infant seat reclined 10 degrees from vertical was used for infant seating. It provided full trunk support via a 15-cm-wide padded foam strap wrapped around the infants' torso and allowed free-range arm and leg movements. A small pillow was used for the head. Before infants could support the weight of their own heads, infants were seated in their caregivers' lap. When transitioned to the infant seat, caregivers sat nearby in another chair. Both MC and ME had already transitioned to the infant seat for collection of the data reported. AC transitioned to the seat at week 19, thus was the only infant who provided data while on her mother's lap.

**Figure 1:**
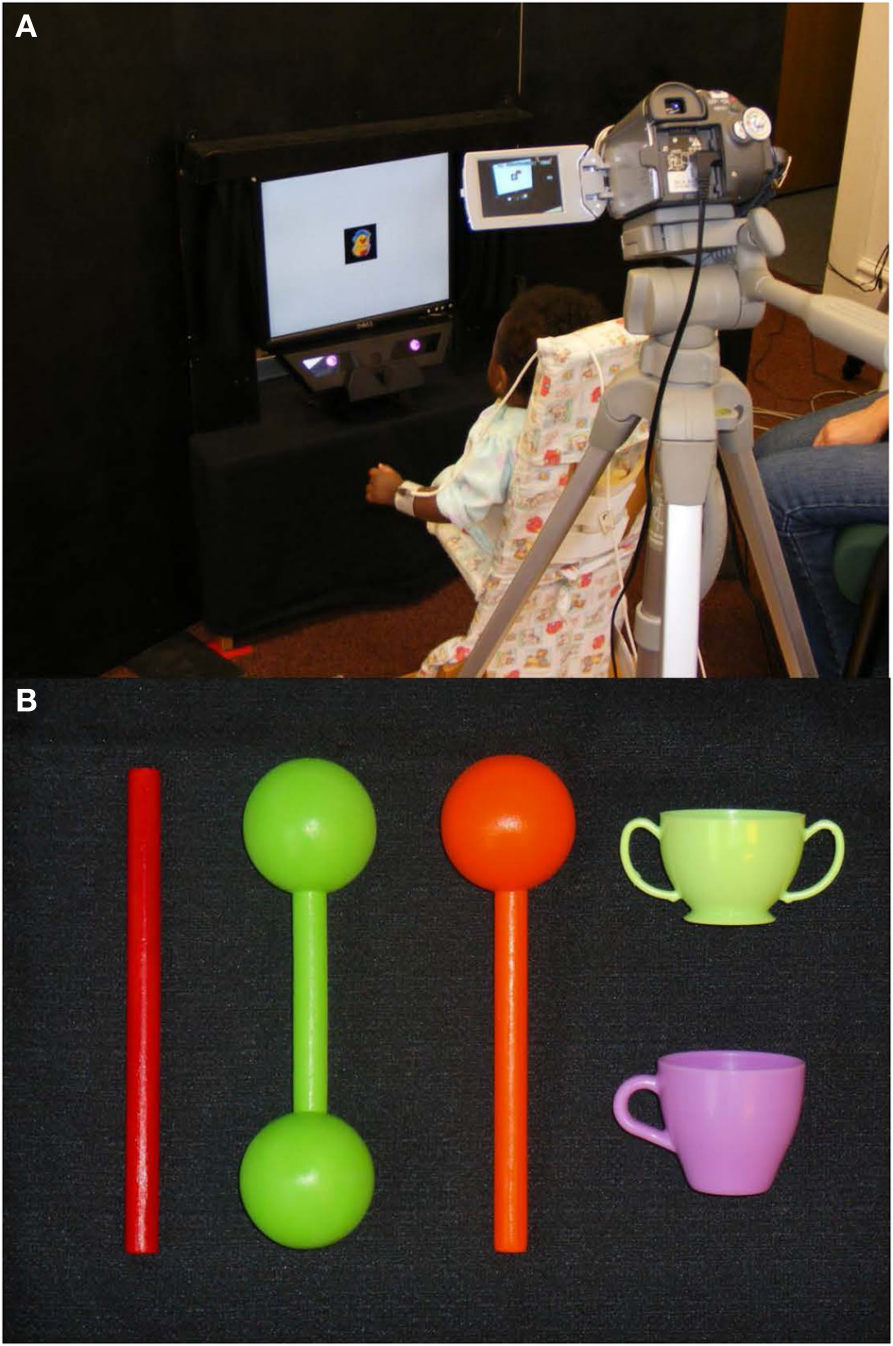
**(A)** Picture of the experimental setup used to track object-directed looking and reaching in infants and **(B)** depiction of the five types of objects used.

To minimize ambient distractions, a custom-designed, black, tri-fold, wooden theater was positioned directly in front of the infants (see Figure 1A). The theater had an opening in the center panel, precisely sized to display a black 15-inch flat-screen monitor mounted on an adjustable arm. The monitor was used for eye calibration. When the flat-screen monitor was removed from the center opening, dual layers of black curtains were positioned to conceal it. A rear curtain, always closed, provided a consistent black backdrop throughout the testing session and concealed the experimenter behind who was presenting the objects to the infants through the opening. The front curtain was opened and closed by this experimenter by using hidden strings located behind the theater in order to reveal the objects.

A Tobii x50 remote eye-tracker (Tobii Technology, Inc., Danderyd, Sweden) was located at the bottom of the presentation window, directly under the flat-screen monitor to capture infants' eye movements during calibration and object presentations. The eye-tracker was positioned at a 60 cm distance from the infants' eyes and its angle was adjusted to accommodate the height of the infants' eyes (usually between 60 and 70 degrees). The eye-tracker, operated through Tobii software (Studio v. 2.0.8), used an infrared light source on the cornea relative to the center of the pupil. Estimated directions of visual fixation and saccade gaze were recorded at a rate of 50 Hz and then were superimposed onto a live video recording of the infants' visual scene, which was captured by a digital camera located directly behind the infant.

Reaching behavior was recorded with three cameras. A small, black webcam facing toward the infant and secured on top of the presentation opening recorded the infants' faces, arms, and hands. This webcam view was merged and saved with the live scene recording containing the infants' looking behaviors. Two additional video cameras were situated on the right and left sides of the infants. They were connected to a Digital Video Switcher (Datavideo Corp., Whittier, CA, USA), which merged the left and right side camera views into one split-screen arrangement and then recorded with an added image frame counter (Horita, Mission Viejo, CA, USA) on a VCR. All camera views, (side reaching cameras, scene camera, and webcam) were synchronized to each other using a small custom-made diodes system (Corbetta et al., 2012).

Infants were offered five different types of objects (see Figure 1B): plain rods (18.5 cm long × 1 cm wide), drumsticks (similar plain rods, 13.5 cm long, with one 5 cm diameter sphere added to one of its ends), dumbbell-shaped objects (made of two 5 cm diameter spheres attached to each ends of a 8.5 cm long rod), small cups (5 × 5 cm with one or two 3 × 1.5 cm handle(s) on the side), and plain spheres (5 cm diameter). The relatively large sizes of these objects were chosen in order to elicit scanning patterns on the objects and enable us to identify if visual selection processes are at work before infants reach for the objects. Most objects were wooden and painted with solid, bright, colorful, non-toxic paint. The cups were made of solid non-toxic plastic. The solid colors ensured that infants would direct attention to the shape of objects. Due to print space constraints, preliminary data from the plain rod and drumstick objects are fully displayed in this report, results for the other objects are discussed in conclusions.

### Procedure

While seated, infants were shown a Sesame Street video (www.sesamestreet.org) playing on the flat-screen monitor positioned in the theater window. When the infant's attention focused on the monitor, the angle of the eye-tracker and the distance between the infant eyes and the eye-tracker were adjusted. Once the capture of the infant's pupils displayed a clear and stable signal, eye calibration using five points began. Calibration points were located at the four corners and center of the monitor. Colorful pictures of objects moving and sounding in concert were displayed consecutively in each of the five areas until the infant had looked at each location for 3–5 s. If any calibration points were missing or inaccurate for either eye, those points were repeated until eye calibration was accurate on at least four out of five points for both eyes. Occasionally, three points were used. When sounds and pictures on the monitor were not sufficient at holding infants' attention to the calibration areas, the experimenter shook small rattles in front of the target areas. Calibration typically lasted between 3 and 10 min.

**Figure 2:**
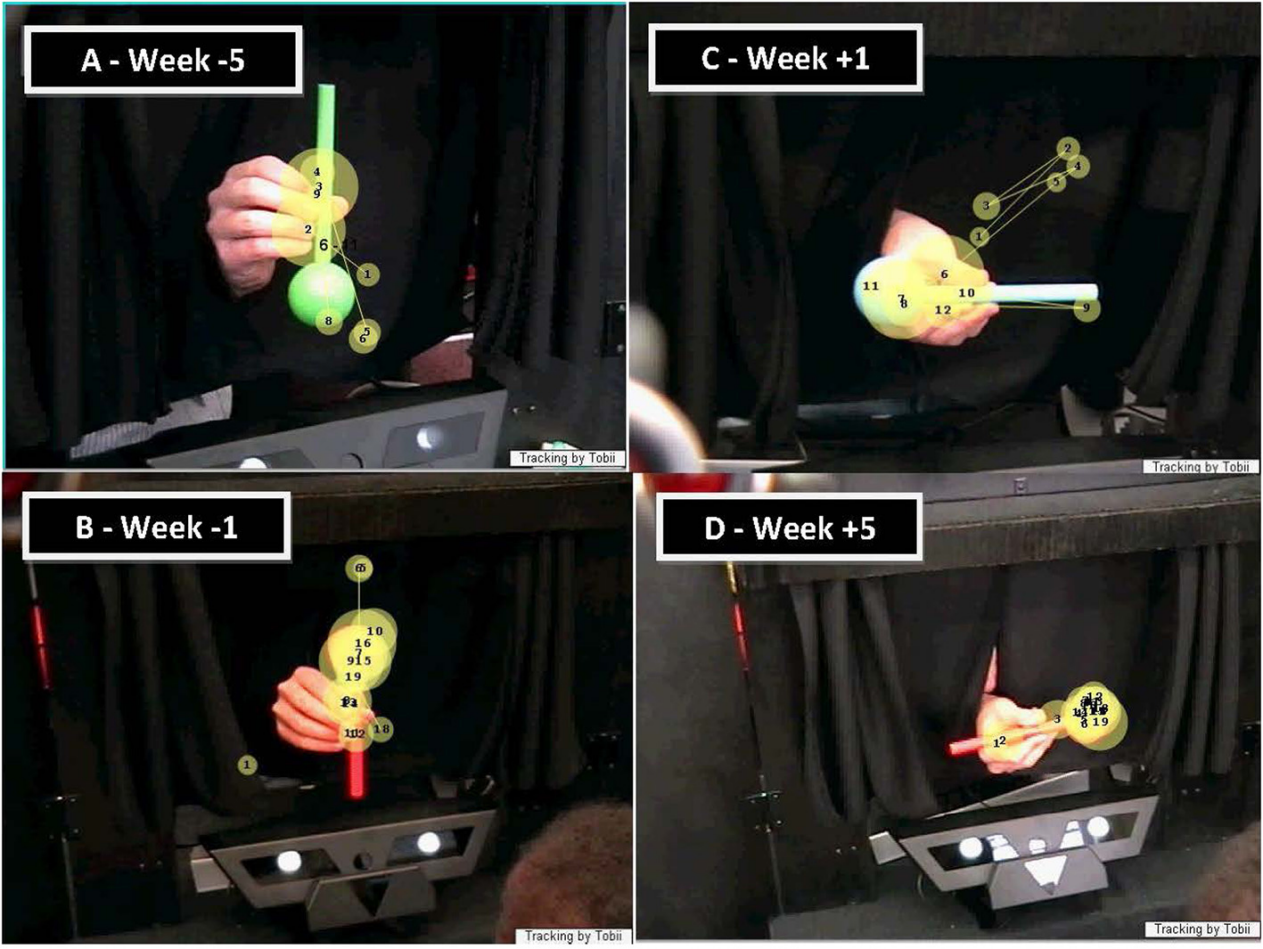
**Illustration of the developmental progression of infant MC's looking patterns directed at the drumstick object**. The object is displayed in all four orientations. The numbered dots on the image indicate the points of fixations, their sizes indicate the duration of the fixations, and the lines correspond to saccades. **(A)** example of object-directed looking pattern performed on week −5 prior to reach onset, **(B)** example of object-directed looking pattern performed on week −1 prior to reach onset, **(C)** example of object-directed looking pattern performed on week +1 following reach onset and **(D)** example of object-directed looking pattern performed on week +5 following reach onset.

After calibration, the monitor was moved out of the infants' view behind the theater, the rear curtain was placed in the back of the open window, and the front curtains were closed to hide the object presentation area. The presenting experimenter sat behind the theater and began each trial by holding an object in place at the center of the calibrated area, right in front of the rear curtain. Once the object was in place, the experimenter gave a verbal signal to a second experimenter located in an adjacent room who was running the eye-tracker. This other experimenter provided an auditory signal when gaze data collection was triggered and the presenter opened the front curtain to reveal the object (see Figures 2A–D). The presenting experimenter, while holding the object steadily in the calibrated window, observed the infant's live gaze on the object from the monitor behind the theater. The object was held out of the infants' reach to approximate as much as possible 5 s of active looking at the scene. Then, the presenter moved the object into the infants' reaching space and the trial ended either when the infant made contact with the object (if capable of reaching), or after a few seconds of holding the object in close arm range to the child (in weeks prior reach onset). If infants reached, they were given 10–15 s to continue touching the object while held by the experimenter (infants cannot grasp objects this young), after which the caregiver took the object away and placed it in a bucket behind the theater out of the infant's view. The next trial proceeded in the same manner.

All objects were presented in both horizontal and vertical orientations. The drumstick had four possible orientations with the sphere located at each one of the four cardinal points while the other objects had two. Each object and orientation were presented twice following a random order, thus the drumstick was presented up to eight times while the other objects up to four times. The same object and orientation were never presented twice consecutively.

### Behavioral coding and analyses

**Figure 3:**
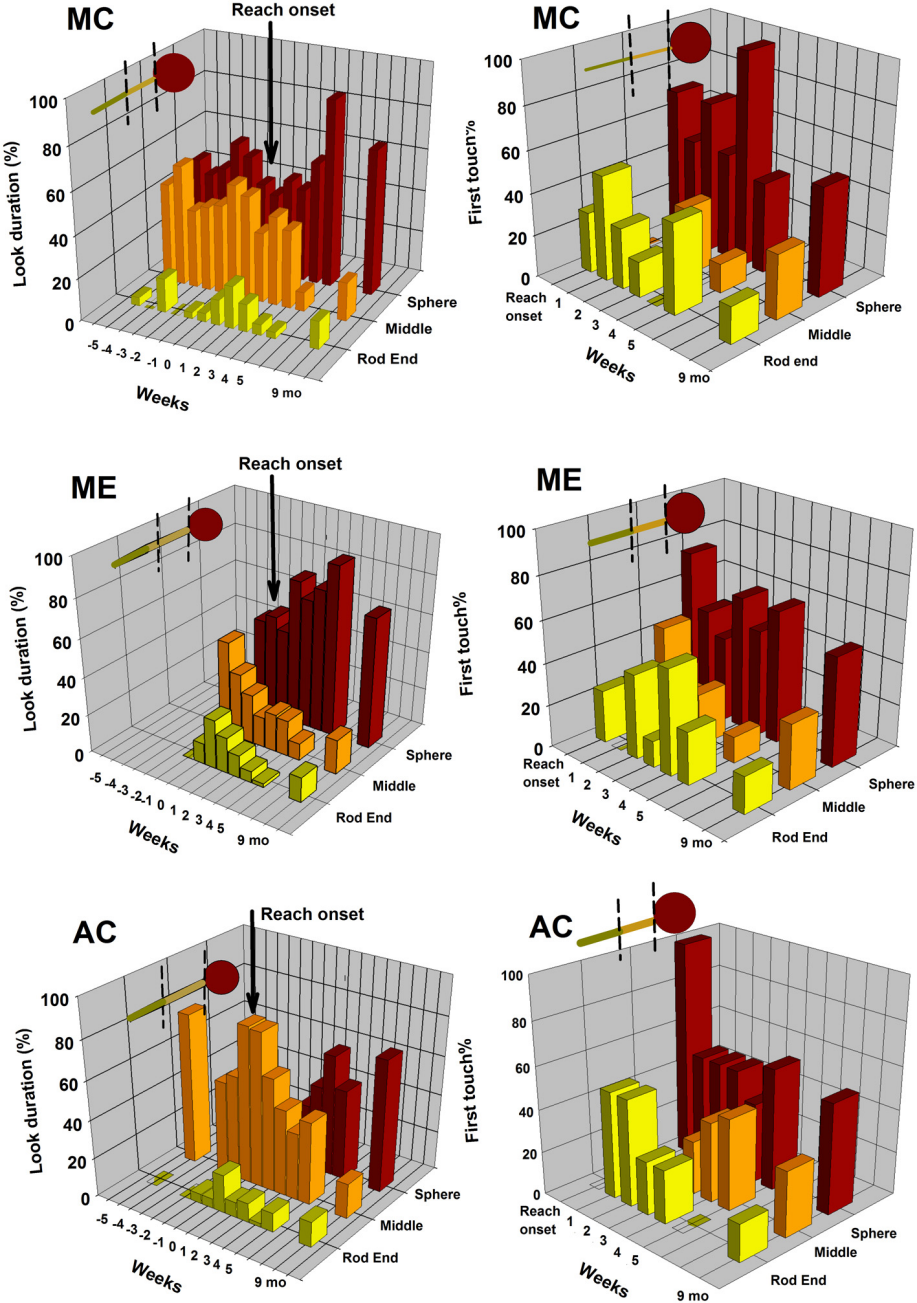
**Drumstick shaped object**. The 3D bar graphs display the distributions of looking patterns on the object (graphs on the left) and distribution of first touched object area (graphs on the right) for each of the three longitudinal infants. Data are plotted by object area (sphere, middle rod, and end rod) and by week of observation. The corresponding results for a group of 9-month-old infants are provided for comparison purposes.

**Figure 4:**
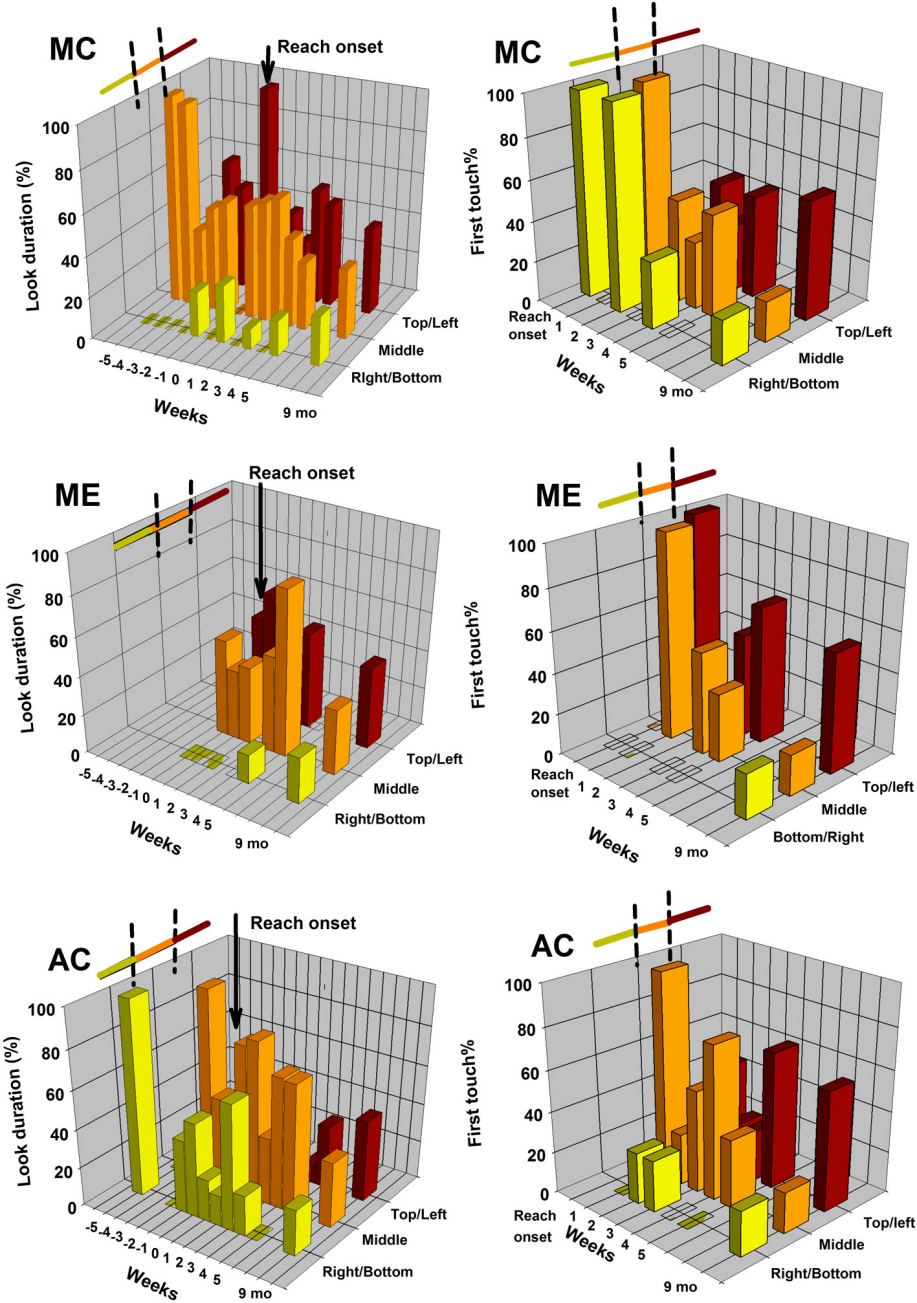
**Plain rod object**. The 3D bar graphs are displaying the distributions of looking patterns on the object (graphs on the left) and distribution of first touched object area (graphs on the right) for each of the three longitudinal infants. Data are plotted by object area (bottom/right end rod, middle rod, and top/left end rod) and by week of observation. The corresponding results for a group of 9-month-old infants are provided for comparison purposes.

All reaching and looking video recordings were imported into and coded in The Observer XT, v 9.0 (Noldus Information Technology Inc., VA, USA). Coding was performed by trained independent observers who identified the onset/offset of fixation points according to predefined regions or areas of interest on the objects and also coded the point of first hand/object contact according to these same predefined object areas (see Figures 3, 4). The coding of looking and reaching were performed independently, in separate passes, to control for possible influences from coding one behavior as a function of the other. Coding of the looking patterns was limited to the time of object exposure in the calibrated window from the moment the curtain opened (revealing the whole object at the center of the theater window) to the moment the presenter began moving the object into the infant's reaching space. Plain rods were divided into three equivalent areas of interest such that when presented horizontally there was a left, middle, and right region and when presented vertically there was a top, middle, and bottom section. The drumsticks' three regions corresponded to a left or right sphere, left or right rod end, and middle rod (when horizontal), or to a top or bottom sphere, top or bottom rod end, and middle rod (when vertical). Looking behavior was coded conservatively by attributing looking to a related object area only when the centers of the fixations were located on the object. Fixation centers located right on the edge of the object were still coded as object-directed fixations, but fixation centers right outside of the object border or located on the hand of the experimenter holding the object were not. We adopted this off-line coding because identifying where the center point of fixation was on the video, specifically in the area where the hand was holding the toy, was easier to determine. Moreover, if the hand holding the toy happened to move slightly, the coders could always and promptly track where the object boundaries were. Finally, the point of hand-object contact, that was coded separately, could later be exported with the looking data in the same spreadsheet. Two dependent measures were extracted from this coding:

- *Looking duration at different object regions*. Looking duration was the accumulated time infants visually attended each pre-defined region of the objects during each object presentation. This coding excluded times when the infant looked at the hand of the experimenter holding the object and when they looked elsewhere on the scene. This duration was normalized as a function of the total looking time on the object during the trial. Inter-observer reliability performed on 20% of the data sample was 93.11% for the longitudinal infants and 91.43% for the 9-month-olds.
- *Location of first hand-object contact*. The location of the first hand-object contact corresponded to the object pre-defined region where it occurred. Inter-observer reliability performed on 20% of the data sample was 80% and 96.7% for the longitudinal and 9-month-old infants, respectively.

#### Description of data corpus

We succeeded at collecting active looking behavior at the scene in all three longitudinal babies within the neighborhood of the 5 s targeted [average overall active looking time per trial and baby in seconds: *MC* = 5.32 (*SD* = 2.51), *ME* = 5.076 (*SD* = 1.80), and *AC* = 8.21 (*SD* = 3.09)]. However, looking behavior was not solely directed at the object, it could be directed at the experimenter's hand holding the object or at the surrounding scene, and, in some trials, infants never looked at the object. We eliminated trials with no or minimal looking at the object, which constituted 13.25% of our data sample, and did not consider looking times that were not directed at the object (i.e., hand and surrounding). Our final data samples and average looking durations at the objects for the longitudinal infants over the 11 weeks used in this report corresponded to: *MC* = 209 trials, object-directed average looking time = 2.51 s (*SD* = 1.45), *ME* = 105 trials, object-directed average looking time = 2.42 s (*SD* = 1.53), and *AC* = 145 trials, object-directed average looking time = 2.76 s (*SD* = 1.57). The drumsticks and rods used for this report constituted 47% of this overall sample. ME and AC produced less object-directed useable data for some weeks preceding reaching onset, which resulted in missing data for those weeks (see Table 1).

#### Statistical analyses strategy

Statistical analyses were focused on capturing trends and developmental changes between periods before and after the onset of reaching within each infant. The strategy adopted was considered the best possible approach given the absence of statistical procedures allowing for the analysis of single subject data. This strategy accounted for the fact that our data are non-parametric normalized proportions, and that all measures are dependent. We first examined if there were predominant looking or reaching behavior at specific object areas as a function of pre- or post-reach onset using a Friedman test. If significant, we followed with pairwise Wilcoxon between object areas to determine where on the objects differences in looking and reaching resided. Development trends within pre- and post-reaching periods were assessed using linear curve estimations on the looking and reaching distributions. To approximate as much as possible an equal number of observations for the weeks prior and the weeks following reaching onset, the pre-reaching period included the 5 weeks before reach onset and the week of reach onset. The post-reaching period included the 5 weeks following reach onset and the week of reach onset. Also, because of low power (analyses performed on 6-week periods at best), we report significance at the 0.05 level, but also *p*-values up to 0.07 level to denote trends toward significance. The 9-month-old data were not included in these longitudinal data analyses. However, we ran Mann–Whitney tests to assess whether the looking and reaching behaviors of the longitudinal infants differed from those of the 9-month-old infants.

## Results

### Looking and reaching at the drumstick

Figure 3 displays the looking and reaching results for the drumstick-shaped object. The 3D bar graphs on the left correspond to the distributions of accumulated looking duration at this object as a function of the three pre-defined object areas (rod end, rod middle, or sphere), the week of testing (−5 to −1 = weeks prior reach onset, 0 = reach onset, 1 to 5 = weeks after reach onset), and infant (MC top graph, ME middle, and AC bottom). The corresponding 3D bar graphs on the right side of this figure display these infants' reaching distributions in relation to where they made first hand contact with the object (rod end, rod middle, or sphere) from the week of reach onset (weeks 0–5). In addition, all six bar graphs display the corresponding data for the group of 9-month-olds for the purpose of comparison. On all graphs, object orientations were collapsed together.

The *p*-values of the statistical analyses performed on these longitudinal data following the strategy outlined above are presented in Tables 2, 4. Table 2 shows that all the Friedman tests that were applied to each of these longitudinal looking and reaching distributional data were significant, meaning that all three infants looked and reached at this object respective pre-defined areas differentially pre- and post-reaching. Wilcoxon tests revealed the following trends. For the *pre-reaching looking period*, both MC and AC divided their object-directed visual attention mainly between the sphere and the middle of the rod. Their amounts of looking at those two areas were significantly greater than at the end of the rod. No test was ran on ME's pre-reaching looking period due to only 2 weeks of useable data up to reach onset. AC's *p*-values for that period were nearing significance. For the *post-reaching looking period*, visual attention to the drumstick was still mainly directed toward the sphere area, middle rod, or both depending on the child. MC's and AC's looking patterns were still mainly distributed between sphere and middle rod, while ME's visual attention was mainly directed to the sphere. Wilcoxon tests performed on the *reaching patterns* indicated a significant bias toward more frequent first touches at the sphere area. All three babies directed their hand and made first contact more frequently with the sphere than the middle rod (significant trend), and end rod (nearing trend). There were no differences in frequency of first touches between middle and end rod areas.

**Table 2:** ***P*-values obtained from the statistical tests applied to (1) the individual distributions of accumulated looking directed to each of the three areas of the drumstick (sphere, middle rod, end rod) for the pre- and post-reaching periods, and, (2) *P*-value of the statistics applied to the individual distributions of the first hand/object contacts**.

**DRUMSTICK**				
**Looking**		**Infant MC**	**Infant ME**	**Infant AC**
**Pre-reaching period**		**(weeks 10–16)**	**(weeks 16–21)**	**(weeks 10–15)**
***N* (weeks)**		**6**	**2**	**4**
**Statistical test**	**Toy areas compared**	***p*-value**	***p*-value**	***p*-value**
Friedman	Sphere vs. middle rod vs. end rod	0.009	–^†^	0.039
Wilcoxon	Sphere = middle rod	ns	–	ns
	Sphere > end rod	0.028	–	0.068
	Middle rod > end rod	0.027	–	0.066
**Looking**		**Infant MC**	**Infant ME**	**Infant AC**
**Post-reaching period**		**(weeks 16–21)**	**(weeks 21–26)**	**(weeks 15–20)**
***N* (weeks)**		**6**	**6**	**6**
**Statistical test**	**Toy areas compared**	***p*-value**	***p*-value**	***p*-value**
Friedman	Sphere vs. middle rod vs. end rod	0.009	0.006	0.032
Wilcoxon	Sphere ≥ middle rod	ns	0.028	ns
	Sphere ≥ end rod	0.027	0.028	ns
	Middle rod ≥ end rod	0.028	ns	0.027
**Reaching**		**Infant MC**	**Infant ME**	**Infant AC**
		**(weeks 16–21)**	**(weeks 21–26)**	**(weeks 15–20)**
***N* (weeks)**		**6**	**6**	**6**
**Statistical test**	**Toy areas compared**	***p*-value**	***p*-value**	***p*-value**
Friedman	Sphere vs. middle rod vs. end rod	0.013	0.041	0.040
Wilcoxon	Sphere > middle rod	0.027	0.042	0.042
	Sphere > end rod	0.066	0.059	0.068
	Middle rod = end rod	ns	ns	ns

ns = p-value > 0.07;

† *no statistics applied for lack of data*.

Developmental trends in *looking behavior* assessed with linear curve estimations (Table 4) only revealed significant changes over time for the post-reaching periods. All three babies did not change looking behavior before reach onset, however, following reach onset, all three babies similarly significantly increased amount of looking at the sphere, while significantly decreasing amount of looking at the middle of the rod. No developmental trends were detected for looking at the end of the rod; this object area continued to be poorly visually attended even after reaching onset. Interestingly, by week 5 after reach onset, the looking patterns distributions at the drumstick in those three infants closely approximated the looking patterns distribution of the 9-month-old group. This older group displayed significantly longer looks at the sphere (Friedman, *p* < 0.0001). The linear curve estimations performed on the *reaching* data did not reveal consistent significant developmental trends across babies, except for AC who increased her object contacts at the middle. For all three babies, the predominant tendency to touch the sphere more frequently remained about the same over the 6 weeks post-reaching period. The 9-month-old infants also displayed significantly more first touches at the sphere (Friedman, *p* < 0.027).

To compare the looking and reaching trends of the longitudinal infants with those of the 9-month-old group, we collapsed the 9-week period (3 weeks before and 6 weeks after reach onset) into three 3-week periods' averages corresponding to: prior to reach onset for looking only (we used week 20 for ME), right after reach onset, and the last 3 weeks post-reaching for looking and reaching. For *looking behavior*, the developmental trends described above were confirmed. The Mann–Whitney tests revealed significant differences between longitudinal and 9-month-old infants for looking at the middle and the sphere areas of the drumstick in the weeks preceding and just following reach onset (sphere prior reach onset, *p* < 0.021, sphere at reach onset, *p* < 0.038, middle rod prior reach onset, *p* < 0.011, middle rod at reach onset, *p* < 0.028, all two-tailed). However, those group differences were no longer significant for the last 3 weeks post-reaching (sphere post-reach, *p* = 0.628, middle rod post-reach, *p* = 0.173, two-tailed). There were no significant differences between groups for looking at the rod end of the drumstick (all two-tailed *p*'s > 0.374). Thus, the longitudinal infants looking patterns at the drumstick, initially different from those of the 9-month-old infants prior to and right around reach onset, became increasingly more similar to those of the 9-month-olds by week 4–6 after reach onset. For *reaching behavior*, there were no significant differences between groups (all two- tailed *p*'s > 0.107), verifying that distribution of the point of first hand/object contact of the longitudinal infants did not differ from those of the 9-month-old group.

In sum, all three longitudinal infants looked and reached at the drumstick differentially over the 11-week period, however, developmental change over time was only observed in relation to the looking pattern performed after reach onset. Infants increased their visual attention toward the sphere, and in a 6-week span, approximated the distributional looking pattern displayed by 9-month-old infants. Interestingly, for reaching, more frequent first contacts at the sphere were present from the week of reach onset. This reaching trend was maintained over the 6 weeks of reaching and was similar to the distribution of points of contacts displayed by the 9-month-old group.

### Looking and reaching at the plain rod

Figure 4 displays the looking and reaching results for the plain rod. As for the drumstick data, the 3D bar graphs on the left correspond to the distributions of accumulated looking duration at this object as a function of the three pre-defined object areas (each end and middle areas). Since there was no specific shape asymmetry to this object, we arbitrarily collapsed the vertical and horizontal presentation trials by merging the amount of looking at the top with the amount of looking at the left, and amount of looking at the bottom with the amount of looking at the right. As for Figure 3, distribution of looking (left graphs) and reaching (right graphs) per object areas are displayed similarly as a function of the week of testing, and by infant following the same order. Again, all six bar graphs display the corresponding looking and reaching data for the group of 9-month-old infants for this same object.

The corresponding *p*-values of the statistical analyses performed on these longitudinal data are presented in Tables 3, 4. Table 3 reveals that very few Friedman tests applied to these individual looking and reaching distributional data reached significance. Thus, as a whole, infants did not display consistent preferred looking biases for the plain rod in the periods preceding and following reach onset, neither did they display consistent biases in reaching. With the exception of MC for the pre-reaching period, and AC for the post-reaching period, infants seemed to have more week-to-week fluctuating looking and fluctuating reaching distributions on the rod with no specific object areas attracting consistently greater looking or reaching behaviors.

**Table 3:** ***P*-values obtained from the statistical tests applied to (1) the individual distributions of accumulated looking directed to each of the three areas of the plain rod (top/left, middle rod, right/bottom rod) for the pre- and post-reaching periods, and, (2) *P*-value of the statistics applied to the individual distributions of the first hand/object contacts**.

**PLAIN ROD**				
**Looking**		**Infant MC**	**Infant ME**	**Infant AC**
**Pre-reaching period**		**(weeks 10–16)**	**(weeks 16–21)**	**(weeks 10–15)**
***N* (weeks)**		**6**	**2**	**4**
**Statistical test**	**Toy areas compared**	***p*-value**	***p*-value**	***p*-value**
Friedman	Top/left vs. middle vs. right/bottom	0.027	–^†^	ns
Wilcoxon	Top/left vs. middle	ns	–	–
	Top/left vs. right/bottom	0.042	–	–
	Middle vs. right/bottom	0.043	–	–
**Looking**		**Infant MC**	**Infant ME**	**Infant AC**
**Post-reaching period**		**(weeks 16–21)**	**(weeks 21–26)**	**(weeks 15–20)**
***N* (weeks)**		**6**	**5**	**6**
**Statistical test**	**Toy areas compared**	***p*-value**	***p*-value**	***p*-value**
Friedman	Top/left vs. middle vs. right/bottom	0.070	0.069	0.030
Wilcoxon	Top/left vs. middle	–	–	0.028
	Top/left vs. right/bottom	–	–	ns
	Middle vs. right/bottom	–	–	ns
**Reaching**		**Infant MC**	**Infant ME**	**Infant AC**
		**(weeks 16–21)**	**(weeks 21–26)**	**(weeks 15–20)**
***N* (weeks)**		**6**	**4**	**5**
**Statistical test**	**Toy areas compared**	***p*-value**	***p*-value**	***p*-value**
Friedman	Top/left vs. middle vs. right/bottom	ns	ns	ns
Wilcoxon	Top/left vs. middle	–	–	–
	Top/left vs. right/bottom	–	–	–
	Middle vs. right/bottom	–	–	–

ns = p-value > 0.07;

† *no statistics applied for lack of data*.

**Table 4:** **Developmental trends in looking distribution and first hand/object contact over the 6 weeks up to reach onset and 6 weeks from reach onset**.

**Drumstick**				**Plain rod**		
**Looking**	**Infant MC**	**Infant ME**	**Infant AC**	**Infant MC**	**Infant ME**	**Infant AC**
**Pre-reaching period**	**(weeks 10–16)**	**(weeks 16–21)**	**(weeks 10–15)**	**(weeks 10–16)**	**(weeks 16–21)**	**(weeks 10–15)**
***N* (weeks)**	**6**	**2**	**4**	**6**	**2**	**4**
**Area of look duration**	***p*-value**	***p*-value**	***p*-value**	***p*-value**	***p*-value**	***p*-value**
Sphere/top-left	ns	–^†^	ns	ns	–^†^	ns
Middle rod	ns	–	ns	0.026	–	ns
End rod/bottom-right	ns	–	ns	ns	–	ns
**Looking**	**Infant MC**	**Infant ME**	**Infant AC**	**Infant MC**	**Infant ME**	**Infant AC**
**Post-reaching period**	**(weeks 16–21)**	**(weeks 21–26)**	**(weeks 15–20)**	**(weeks 16–21)**	**(weeks 21–26)**	**(weeks 15–20)**
***N* (weeks)**	**6**	**6**	**6**	**6**	**5**	**6**
**Area of look duration**	***p*-value**	***p*-value**	***p*-value**	***p*-value**	***p*-value**	***p*-value**
Sphere/top-Left	0.047	0.023	0.033	ns	ns	ns
Middle rod	0.035	0.019	0.009	ns	ns	ns
End rod/bottom-right	ns	ns	ns	ns	ns	ns
**Reaching**	**Infant MC**	**Infant ME**	**Infant AC**	**Infant MC**	**Infant ME**	**Infant AC**
	**(weeks 16–21)**	**(weeks 21–26)**	**(weeks 15–20)**	**(weeks 16–21)**	**(weeks 21–26)**	**(weeks 15–20)**
***N* (weeks)**	**6**	**6**	**4**	**6**	**4**	**5**
**Area of look duration**	***p*-value**	***p*-value**	***p*-value**	***p*-value**	***p*-value**	***p*-value**
Sphere/top-left	ns	ns	ns	0.032	ns	ns
Middle rod	ns	ns	0.006	ns	ns	ns
End rod/bottom-right	ns	ns	ns	ns	–^‡^	ns

*P-values from linear trend testing are displayed by object area and by infants*.

† no statistics applied for lack of data;

‡ *no look at the bottom/right end rod*.

Likewise, Table 4 shows that the linear curve estimations applied to these data revealed almost no developmental trends over the 6-week periods preceding and following the emergence of reaching. The only significant linear change over time observed in looking was for infant MC, who reduced visual attention to the middle of the rod during the pre-reaching period. Also, MC was the only one to display a significant linear change in reaching; she increased her amount of first hand contact with the top/left of the rod over the 5 weeks following reach onset.

In sum, compared to the drumstick that yielded looking and reaching patterns that seemed to gravitate predominantly toward the sphere in all three infants, the plain rod seemed to entice more random trends. Note that for the 9-month-old infants, looking patterns on the rod were also more distributed across all three object pre-defined areas (Friedman, *p* = 0.891). Reaching, in that older age group was biased toward the top/left rod area (Friedman, *p* < 0.027). A similar trend can be seen in the longitudinal infants, although it is present only for week 5 post-reaching. None of the group comparisons between longitudinal and 9-month-old infants revealed significant difference in looking and reaching behavior for any of the object areas and any of the collapsed 3-week periods.

### Visual-motor mapping following the onset of reaching

**Figure 5:**
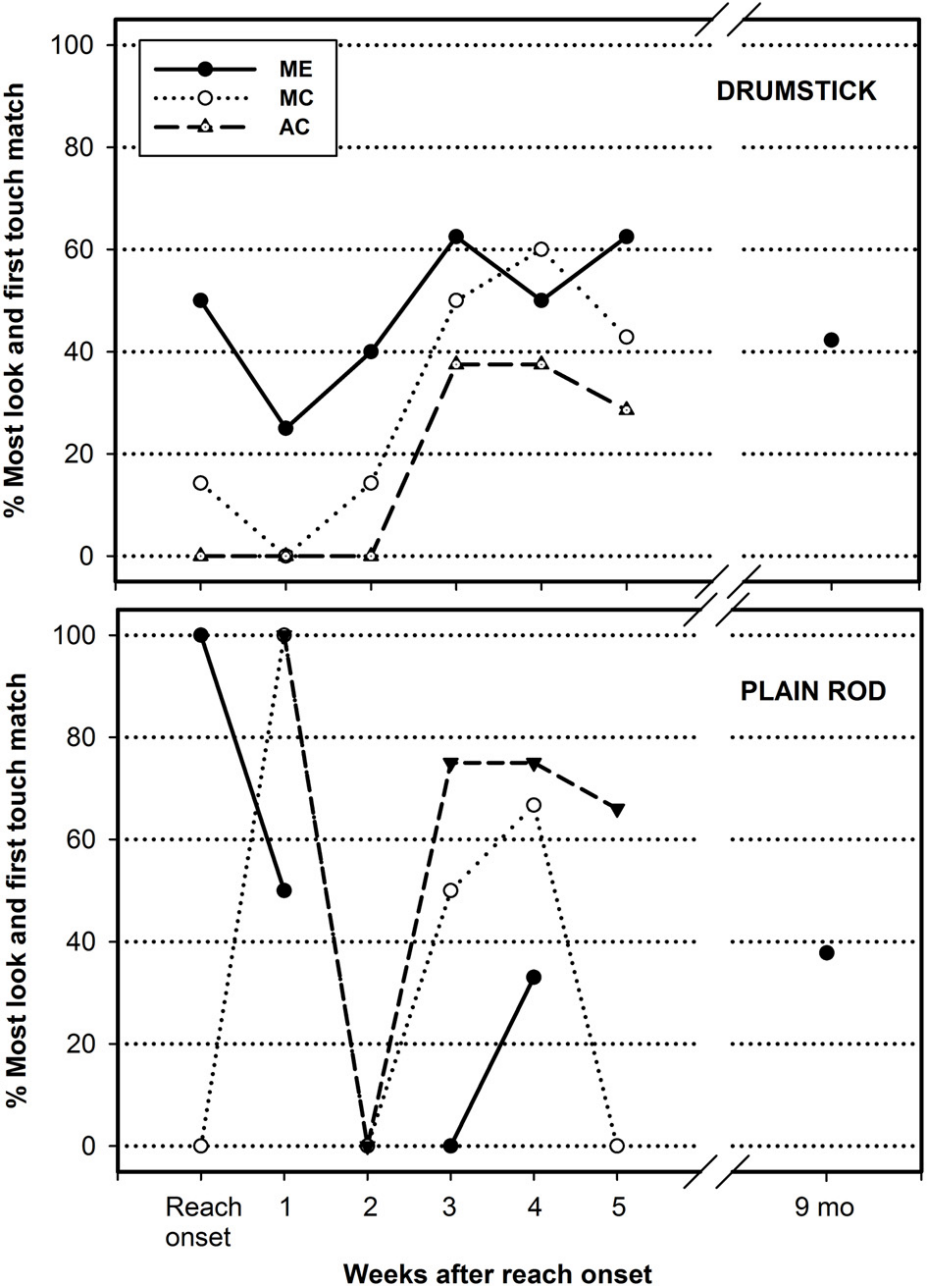
**Rate of within trial matches between the most looked object area and the first touched object area by infant, by object (drumstick top graph, plain rod bottom graph) and by week following reach onset**. The corresponding results for a group of 9-month-old infants are provided for comparison purposes.

The data presented above reflected changes in looking and reaching behaviors independently. To address the question of the mapping between the feel of the hand and the sight of the object, looking and reaching behaviors needed to be linked to each other. To address this, we performed a trial-by-trial analysis to examine whether there was a direct spatial correspondence between the areas of the object visually attended the most (the most looked area) and the location where the hand made the first contact with the object (area of first touch). The number of trials corresponding to a direct spatial match between the most looked area and the area of first hand contact were normalized as a function of the total number of trials collected for a given object. These data are reported in Figure 5 by week from reach onset, and for each longitudinal infant separately, drumstick on top and plain rod at the bottom. These same data for the 9-month-old group are displayed on these graphs for the purpose of comparison.

We performed a linear curve estimation on these data to assess changes in the rate of spatial look-reach match over time. For the drumstick, the rate of matching between looking and reaching revealed a 2 to 3-fold increase over the observed 6-week period, but reached significance only for AC (*p* < 0.048). It neared significance for MC (*p* = 0.055) and was not significant for ME (*p* = 0.209). For the plain rod, there was no significant developmental trend observed. Mann–Whitney tests comparing the 3-week averages of the longitudinal infants with the data of 9 months old group revealed a nearing group difference (*p* = 0.065) for the first 3 weeks following reach onset in the drumstick. All other comparisons (last 3 weeks for drumstick and plain rod group comparisons) were not significant (all *p*'s > 0.244).

The last analysis assessed where on the object the look-reach match occurred to determine if visuo-motor matches occurred randomly on any areas of the object or if they were focalized to one or two specific areas of the objects. To do so, we considered only the trials that yielded a look-reach spatial match. For the drumstick, 88% of the look-reach matches documented over the 6-week period were aimed at the sphere area of the object, the remaining 12% occurred at the middle rod area. There were no look-reach matches corresponding to the end of the rod for this object. For the plain rod, 77% of the matches occurred at the middle of the rod, the remaining 23% percent was spread at either end areas of the rod. These numbers are combining all three infants and all weeks. These trends indicate that when spatial matches occurred between looking and reaching, they did not occur randomly on either area of the object, but were mainly focused on the sphere area for the drumstick and the middle area for the plain rod. These trends were present from the first weeks of reaching in all three infants and lasted up to the last week of reaching reported.

## Discussion

The goal of this paper was to begin evaluating how infants map the feel of their arm with the sight of the object when they perform their first goal-directed reaching movements. To examine this mapping process, we tracked the object-directed looking behavior (via eye-tracking) and point of first hand-object contact in three infants that we followed weekly over an 11-week period throughout the transition to reaching. With these data, we evaluated three possible scenarios that offered different levels of balance between the respective roles that vision and proprioception could have at the emergence of infant's reaching. The prospective control hypothesis assumed a more predominant role of vision over proprioception, the embodied hypothesis assumed a more predominant role of proprioception or sensory-motor experience over vision, and finally, a more mutually balanced contribution of both vision and proprioception acting in concert was considered as a third possible hypothesis. For each hypothesis, we made specific predictions. Here we discuss our findings against these predictions.

- *Scenario 1 (or the prospective control hypothesis)*. This scenario assumed that vision would develop its prospective control role prior to reach onset. Reaching would form as a result of the infants increasingly figuring out how to proprioceptively guide their hand toward the area where visual attention is directed. Predictions consistent with this scenario were that (1) vision would reveal specific selective looking trends at the objects in the weeks preceding reach onset, (2) that after reach onset the looking trends would persist, and (3) that change over time would occur in the reaching behavior as a result of the more successful spatial alignment (or mapping) of the action endpoint onto the visually selected object area. For the drumstick, we observed a looking bias prior to reach onset directed toward two object areas—the sphere and the middle rod areas. This looking bias grew more specific in the direction of the sphere after reach onset, while all three infants maintained a relatively steady reaching bias at the sphere from reach onset and thereafter. Thus, for this object, a developmental change was observed in the looking behavior following reach onset but not in the reaching behavior *per se*, which is inconsistent with this scenario's predictions. Similar trends were not observed for the plain rod. In fact, very few significant results were reported for this plain rod object, suggesting that object shape may have interacted with this perceptual-motor mapping process, a point discussed further below, also in relation to the other objects that we did not present.
- *Scenario 2 (or the embodied account hypothesis)*. This scenario assumed that infants build an extended, proprioceptive based sense of where to move their arm in space through initially undirected, nonetheless active movement experiences prior to reach onset. As infants happen to touch objects in their surroundings, at first accidentally (and without looking), they discover how to connect their visual attention to the consequential proprioceptive and haptic feel of their arm and hand in the environment. This would allow the formation of some kind of proprioceptive knowledge of how to direct arm movements in space by associating spatial vision to movement perception, leading to reach onset. The predictions we made in relation to this scenario were that infants (1) would not necessarily show specific object-related visual trends prior to reach onset, but (2) would demonstrate a more accurate/consistent spatial aiming ability in their reaching movement from reach onset, as a result of their acquired proprioceptive spatial movement experience in the weeks preceding reach onset. We also expected a change in visual attention toward the touched area (rather than a change in movement aiming toward the looked area) after reach onset as a product of successful reaching. The drumstick results were in line with these predictions. All three infants displayed a greater first touch trend at the sphere from reach onset. They maintained this aiming trend over the post-reaching weeks. Developmental change occurred in the looking pattern after reach onset. Over the post-reaching weeks, visual attention increasingly shifted toward the sphere, the preferred first touch area. Again, similar trends were not observed for the plain rod.
- *Scenario 3 (co-mapping of sight and feel)*. This scenario assumed that both vision and action would map onto each other following reach onset. With this scenario, we did not predict a dominant trend in looking prior to reaching nor did we predict a dominant trend in reaching from reach onset. Rather, we expected initial random looking and reaching patterns that would reciprocally map onto one another over time. Both vision and reaching would adjust and morph into a more precise perceptual-motor mapping over time. Infants would increasingly try to look to where they aim, while trying to aim to where they look at the same time. None of our data revealed concomitant changes in both looking and reaching over the observed developmental period to support this third scenario.

### Possible implications for the process of learning to reach and the development of prospective control in infancy

The looking and reaching behaviors documented through the transition of reaching in those three infants seem to point toward the embodied hypothesis, but as noted, this was only for the drumstick-shaped object; these findings did not extend to the plain rod object. Here, we discuss these results in relation to the other objects that we have not presented and evaluate the significance and limitations of these preliminary findings for our understanding of visuo-motor mapping in infants learning to reach.

We found the trend reported for the drumstick at first provocative. The long held belief in the infant reaching literature (even from prior embodied accounts) has been that infants have poor control of their arm at reach onset, hence the documented indirect trajectories (Thelen et al., 1993, 1996; von Hofsten, 1991). As a result, assumptions were that, from reach onset, infants first learned how to bring their arm more successfully in contact with the object and only after extensive practice, did they learn to refine their arm control to direct their hand more accurately and more smoothly toward the target taking into account its physical characteristics (Lockman et al., 1984; von Hofsten and Fazel-Zandy, 1984). According to such assumptions, we would have expected more random points of first hand-object contacts for any object following reach onset. The fact that, for the drumstick (and for the other objects also, as we will see), all three infants succeeded touching the sphere more predominantly from the beginning, suggests that infants are somewhat capable of aiming their movement in space more accurately than thought before. This result is particularly striking given that the sphere orientation was randomly presented in one of four possible cardinal locations. Thus, to touch the sphere first more often and from the first week of reaching, the infants had to have developed some basic control ability to direct their arm to those different locations in space. Related to this finding was the fact that the observed increase in look-reach match also occurred predominantly at the sphere area, not at other object areas, and that this match increase seemed to result more from an augmented visual attention toward the sphere across weeks, not a change in touch rate at the sphere.

If we think a little more about this result on reaching accuracy, we realize that it may not be so unexpected after all. Prior studies on infant reaching have typically used small objects for reaching and shown that at reach onset infants can hit such smaller objects. Thus, in a way, prior studies have already demonstrated that infants are capable of some spatial movement accuracy. However, this ability never came into clear focus, possibly because no studies had observed how infants could begin reaching for larger objects offering choices in points of contact.

We also think that the object shapes and spatial arrangement of their distinct features mattered in driving the responses observed. When spheres or larger parts were present (as in the drumsticks, dumbbells, or cups), the looking and reaching responses were more skewed toward the sphere(s) or cup bowl. Skewed responses appeared stronger when the bigger part of the object was one, as in the drumsticks or cups. For the cups, for instance, looking and reaching were heavily directed at the bowl of the cup, not the handle(s). This trend for the cups was also present in the 9-month- old group (there were no significant differences between groups). Shape features also seemed to engender more developmental changes (as we saw for the drumstick). For example, for the dumbbell-shaped object, two out of the three infants (MC and ME) displayed growing visual attention toward the two spheres located at each end of the rod in the post-reaching weeks compared to the pre-reaching weeks. This developmental change was not as strong as the one reported for the drumstick shaped object due to the fact that, for the dumbbell, visual attention was being increasingly split between two sphere locations (instead of one as in the drumstick). For MC, the sphere/middle rod/sphere looking distributions for the dumbbell went from 24/76/0 on week −5 to 48/13/39% on week 5. For ME, the pattern distribution was 66/34/00 at week −1 and was 45/0/55% at week 5. The looking distributions at week 5 for the dumbbell were not significantly different from those of the 9-month-old group (41/18/41%). Reaching, on the other hand, was already directed toward one of the two spheres more frequently from reach onset (MC sphere/middle/sphere percent reaching = 71/2/27% at reach onset and 25/0/75% at week 5 post-onset, ME sphere/middle/sphere percent reaching = 6/35/59% at reach onset and 75/0/25% at week 5 post-onset; 9-month-olds = 46/18/36%). Finally, as a result of two visually attended areas, but only one touched area, the rate of look-reach match for the dumbbell was not showing as a consistent progression over time as reported for the drumstick with one sphere. But this low rate of look-reach match was not void of trends. We reported that for the drumstick, when matches between looking and reaching occurred, they occurred in great majority at the sphere location. For the plain rods, even though there was no strong, consistent progression between looking and reaching matches, when matches occurred, they happened at the middle of the rod area. The same area of match consistency was found for the dumbbell and cup objects. For the dumbbell, when look and reach spatially matched, they occurred 80% of the time on one of the two spheres (only 20% of the matches were performed in the middle rod area), and for the cups, 94% of the look-reach matches occurred at the bowl area (again, these trends are consistent with what we observed with the 9-month-olds). Thus, from all of the above, it appearss that object shape drove infants' reaching responses and visual attention differentially, otherwise we would not have obtained such response trends and regularities within and across objects. Furthermore, a steady reaching trend from reach onset was observed for nearly all objects when distinct shape features were present, while it was not always the case for looking.

We also did not expect infants showing points of object contact so similar to those of the 9-month-olds right from reach onset. And we did not expect infants' looking patterns to change so quickly to resemble those of the 9-month-olds in just a few weeks. These findings were surprising but also suggest clues to our understanding of the process of visuo-motor mapping at reach onset.

First, we think that these data show that from reach onset infants can project their hand toward a future location in space successfully and can display a certain level of endpoint accuracy, similar to those of more experienced reachers. This supports the interpretation that, infants must have developed some kind of proprioceptive spatial knowledge of their arm movement prior to learning to reach. As discussed earlier, such motor knowledge could have formed as a consequence of accidental events involving the arm and the eyes, which possibly created useful visuo-motor contingencies that helped the development of an extended sense of the arm movement in space (see also Borghi et al., 2013 on the concept of extended embodied mind). We know that blind infants who cannot use vision to spatially calibrate their actions in space are delayed in their development of goal-directed skills (Bigelow, 1986), while infants who have had visual experience of the world prior to reach onset can begin reaching in the dark, even at first with minimal visual information (Clifton et al., 1993). We think that while the formers may be deprived from building such extended proprioceptive mapping of their actions in space as a result of their lack of vision, the latters benefit from being able to associate visual experience to their movement experience, thus, allowing them to reach successfully in the dark in response to a seen target, but also in response to auditory cues (Clifton et al., 1991). Clearly, given our procedure, and the fact that we gave time to the infants to look at the objects prior to allowing them to reach for them, we cannot rule out that by doing so, we may have enhanced their visual attention to the objects, which could possibly explain the selective object-related responses we observed. It is possible, that with such object attention enhancement, we allowed infants to consider the spatial properties of the objects more fully than if they were not given time to look at the object prior to aiming for them. Obviously, the present results need to be substantiated on a larger sample and extended to other task contexts to fully understand the underlying processes of early perceptual-motor matching. But, the fact that all three infants in our setup displayed similar trends on many of these measures is remarkable in our opinion.

Second, the fact that for the drumstick the greatest point of hand-object contact seemed more consistent over the weeks, while the point of greatest visual attention grew to align with the point of hand-object contact over the weeks, suggests that vision may not have been the main driving factor in setting initial motor goal accuracy. However, as spatial mapping between vision and action strengthened over the weeks, vision may have become more predictive in defining the point of where to bring the hand in contact with the object. Again, this was suggested by the progressive alignment of vision onto the preferred contacted area for the drumstick. Such alignment could reflect an increasing ability of vision to become more selective and more predictive of where the hand is being directed as motor and visual spatial outcomes are being paired repeatedly during early reaching responses. The prospective role of vision could originate from these infants' initial embodied reaching experiences. Indeed, it could be possible that infants' movement experience and associated resulting action's outcomes drove the needs of vision to begin detecting ahead of time where the hand should go. In the case of reaching for the drumstick, infants could have learned to direct their visual attention increasingly toward the sphere area, perhaps because it met some valued outcome. For example, infants may have preferred to touch the sphere because it provided greater haptic experience than the thinner rod to which the sphere was attached. As infants gained experience at reaching and touching, vision became increasingly attuned to these features and began performing more searches for these special features, thereby becoming more selective and predictive for reaching. Such interpretation is consistent with a number of studies on infants' self-produced actions and their understanding of actions in the physical and social world that suggest, in similar ways, that infants' active experiences can drive changes in their attention and perception of the world (Cicchino and Rakison, 2008; Rakison and Krogh, 2012). Another study also found that infants' observational experience of others' actions does not lead to the same understanding as when acting themselves (Gerson and Woodward, 2014). Thus, findings from these studies are consistent with our stand that vision alone, may initially not provide the best source of information in the context of goal-directed actions, but experience acquired through early sensory-motor activity may foster a discovery and understanding of the world that could eventually translate into a more cognitive or visual knowledge of the world (see also, Campos et al., 2000 on motor activity and mind).

Future studies are necessary to extend our observations to more infants and wider contexts to examine the validity of the embodied scenario we propose. Most useful, we think, will be studies examining vision during the movement of reaching itself, something we did not do in our longitudinal observations. Such observations will be essential to disentangle the respective role of vision and arm control in infants' first reaching attempts. Prior evidence, in 9-month-old infants, where the recording of infants' eye-movements directed to a target were paired with the arm movement kinematics corresponding to reaching for that same object, pointed to the production of object-specific looking patterns closely matching movement corrections toward that object (Corbetta et al., 2012). It is unknown whether infants at reach onset can perform such eye-hand corrections during movement. Detecting whether such on-line attentional patterns and movement corrections also occur in young infants at reach onset will be important to continue to understand how infants discover how to map the feel of their arm with the sight of the object.

### Conflict of interest statement

The authors declare that the research was conducted in the absence of any commercial or financial relationships that could be construed as a potential conflict of interest.
